# Anesthesia, the developing brain, and dexmedetomidine for neuroprotection

**DOI:** 10.3389/fneur.2023.1150135

**Published:** 2023-06-07

**Authors:** Alexandra Tsivitis, Ashley Wang, Jasper Murphy, Ayesha Khan, Zhaosheng Jin, Robert Moore, Vahe Tateosian, Sergio Bergese

**Affiliations:** ^1^Department of Anesthesiology, Stony Brook University Hospital, Stony Brook, New York, NY, United States; ^2^Renaissance School of Medicine, Stony Brook University, Stony Brook, New York, NY, United States

**Keywords:** dexmedetomidine, neuroprotection, neuroplasticity, postoperative delirium, anesthetic neurotoxicity

## Abstract

Anesthesia-induced neurotoxicity is a set of unfavorable adverse effects on central or peripheral nervous systems associated with administration of anesthesia. Several animal model studies from the early 2000’s, from rodents to non-human primates, have shown that general anesthetics cause neuroapoptosis and impairment in neurodevelopment. It has been difficult to translate this evidence to clinical practice. However, some studies suggest lasting behavioral effects in humans due to early anesthesia exposure. Dexmedetomidine is a sedative and analgesic with agonist activities on the alpha-2 (ɑ_2_) adrenoceptors as well as imidazoline type 2 (I2) receptors, allowing it to affect intracellular signaling and modulate cellular processes. In addition to being easily delivered, distributed, and eliminated from the body, dexmedetomidine stands out for its ability to offer neuroprotection against apoptosis, ischemia, and inflammation while preserving neuroplasticity, as demonstrated through many animal studies. This property puts dexmedetomidine in the unique position as an anesthetic that may circumvent the neurotoxicity potentially associated with anesthesia.

## 1. Introduction

Neurotoxicity can be defined as any unfavorable effect on the central or peripheral nervous systems’ chemistry, structure, or function induced by chemical or physical agents either at maturity or during development (1).

There is widespread, replicable evidence that anesthetic agents cause neurotoxicity in a variety of animal models. However, translating this research to clinical practice is difficult given the ethical limits of human experimentation and the multitude of confounding factors of cohort studies. Despite these limitations, this animal evidence has led to obvious concerns about pernicious effects of anesthetic agents on the central nervous system. These include pediatric neurotoxicity and post-operative delirium in both children and the elderly. Human studies offer mixed results: some show lasting behavioral effects of early anesthesia exposure and others report no consequences (2–5). Disagreement in the literature has caused anesthesia induced neurotoxicity to become the subject of heated and ongoing debate.

Over the last 20 years researchers in anesthesia neurotoxicity have had several goals: to demonstrate causation, identify high risk groups, uncover cellular mechanisms of damage, and find neuroprotective compounds which mitigate toxicity. Dexmedetomidine might be one such agent. This review seeks to clarify possible mechanisms and existing evidence about the use of dexmedetomidine to migrate potential anesthetic neurotoxicity.

Dexmedetomidine is a widely used sedative and analgesic that acts through agonism of alpha-2 (ɑ2) adrenoceptors (6, 7) as well as imidazoline type 2 (I2) receptors (8), allowing it to offer possible protective effects in the nervous system against apoptosis, ischemia, and inflammation (8). Given its neuroprotective potential, dexmedetomidine may present itself as an alternative to other typical anesthetics and a probable solution to anesthesia neurotoxicity.

Dexmedetomidine has been suggested as an agent to prevent Postoperative delirium (POD) in elderly patients. POD is a syndrome characterized by fluctuating acute cognitive disorder, confusion, restlessness, and agitation after anesthesia, known to affect elderly populations (9–11). Clinically, it may present as hyperactive, hypoactive, or mixed during its course, and is a known serious complication to general anesthesia in the elderly. Recent studies show the use of dexmedetomidine as a potential treatment to reduce POD by lowering post-operative pain and opioid use (9, 12). The application of dexmedetomidine to prevent POD in elderly patients has been studied and reviewed in orthopedic, cardiac, oncologic, and noncardiac major surgery. In general, there is a great deal of heterogeneity in clinical studies related to the use of dexmedetomidine to prevent POD. Existing studies may assist for specific clinical choices but there is insufficient evidence to support broad conclusions and application. Overall, there are numerous areas for thoughtful and impactful research.

Dexmedetomidine has numerous clinical applications in the pediatric population including treating emergence delirium and agitation. Eight recent studies discuss the impact dexmedetomidine has towards emergence delirium (ED), a condition of hyperarousal and mental disorientation marked by a state of hyperexcitability during general anesthesia recovery (13) and emergence agitation (EA), an uncomfortable state of intense arousal, in pediatric populations (14). The applications are discussed and measured using primarily the pediatric anesthesia emergence delirium scale (PAEDS), which has been widely accepted as diagnostic criteria for emergence delirium in pediatric populations undergoing general anesthesia (15). These studies may suggest real world benefits via the reduction of maladaptive CNS impacts of other anesthetic agents.

### 1.1. Search strategy

Authors identified and selected articles to review using the most updated data, ranging from 1991–2022. Articles were identified via the use of PubMed, Central, EMBASE, CINAHL, Google Scholar, and the Web of Science citation index Search terms included: anesthesia neurotoxicity; dexmedetomidine Neuroprotection; Post-operative Delirium; emergence delirium; and emergence agitation Articles describing: clinical literature reviews; basic research reports; clinical trials; case reports; systematic reviews, and meta analyses were considered eligible for review.

## 2. Anesthesia neurotoxicity

### 2.1. Animal model studies

Todorovic et al. conducted one of the first seminal studies that investigated anesthesia-induced neurotoxicity using a rat model (16). After exposing 7 day-old rats to common anesthetic agents (nitrous oxide, isoflurane, and midazolam), Todorovic et al. found widespread neuronal apoptosis with severe neurological defects in the infant rats. These results raised serious concerns about the consequences of anesthesia exposure in the pediatric population, sparking a slew of experiments that examine the neurotoxic effects of anesthetics in rodent models. Subsequent studies supported Todorovic et al.’s findings, showing higher rates of neuroapoptosis in the developing rodent brain upon exposure to other anesthetic compounds such as ketamine (17) and propofol (18). Rodent models have also provided insight into the cellular mechanisms of anesthesia neurotoxicity. The damage caused by anesthesia exposure seems to affect many different biochemical processes that may lead to iron overload (19), disruption of mitochondrial fission and fusion (20), dysregulation of calcium (21), activation of the endoplasmic reticulum stress pathway (22), BDNF-dependent neuroapoptosis (23), as well as other processes (24).

The similarities in architecture and development of rat and human brains make the rodent model a powerful tool for such studies (25). However, there are also notable differences, such as the length of brain growth spurt (years in humans versus weeks in rodents) and gyrification (the development of folding patterns of sulci and gyri on the brain surface, which are present in humans but largely absent in rodents) (26). These differences highlight the need for more representative animal models in studying anesthesia-induced neurotoxicity. Since brain development and behavior of primates are more analogous to humans, the use of non-human primate models offers a new avenue to better understand anesthetic-induced neurotoxicity in the nervous system.

Studies of anesthesia neurotoxicity in rhesus macaques have reaffirmed the neuroapoptosis already observed in rodents upon anesthesia exposure. These studies tested a range of anesthetics such as propofol (27), isoflurane (28), and ketamine (29). All reached the conclusion that anesthesia exposure during brain development results in significant neuronal and glial apoptosis when compared to non-exposed animals. Furthermore, studies using non-human primate models have also examined the potential long-term behavioral or psychiatric effects of anesthesia exposure during early brain development. A 2017 study by Alvarado et al. tested the effects of repeated exposure to sevoflurane on visual memory in newborn rhesus monkeys (30). They found that exposure to sevoflurane during neonatal periods was associated with statistically significant visual memory impairment at age 12 months and older. Similarly, Paule et al. found lower performance in cognitive function tests (e.g., learning, color discrimination, and short-term memory) in 10 month-old rhesus monkeys that endured 24 h exposure to ketamine as neonates compared to their non-exposed counterparts (31). These results seem to suggest that early exposure to anesthesia may have negative, long-lasting consequences in the neurological function of non-human primates.

When evaluating animal studies, one must be mindful of the methods employed. For example, the high level of anesthetic exposure to newborn animals may not be representative of the experience of most pediatric patients undergoing anesthesia in the context of routine clinical practice. Two studies by Coleman et al. (32) and Neudecker et al. (33) specifically examined the long-term cognitive effect of one- versus three-time exposure to isoflurane during the neonatal period in monkeys, noting an exposure-dependent relationship to phenotype. Coleman et al. found that the multiple exposure monkeys demonstrated motor deficits, increased anxiety, and affiliative behavior, where no significant deficits or changes were found in the single exposure group (32). Neudecker et al. utilized cognitive function testing and behavioral assessment, reporting no changes in cognitive function in either exposure group relative to the non-exposed control (33).

Although rhesus monkeys are developmentally, genetically, and physiologically (34) similar to humans, there are several limitations to consider with animal models. For one, the development and social factors of non-human primates must be tightly controlled to ensure proper comparison between control and test groups. Additionally, animals undergoing trials are generally in healthy conditions, whereas pediatric surgery patients may be in poor health with other significant comorbidities. Finally, one should understand that anesthesia exposure in animal model studies occurred in absence of surgery. In contrast, anesthesia and surgery are almost always associated in clinical practice, wherein surgery itself may impact the developing human.

### 2.2. Human studies

Translating animal results to human studies has proven to be difficult. For obvious ethical reasons, the experiments demonstrating causation in animal models cannot be done in human research. The bulk of human research in anesthesia neurotoxicity has been through observational studies, of which there are over 90 published as of 2019 (35). Many of these studies report conflicting results. Lack of consensus could be due to potential confounding factors associated with observational studies, such as including children with congenital abnormalities or intraoperative complications.

Most of these observational studies identified populations of children, and compared measurements of neurocognitive outcomes between children who had received early-life anesthesia and those that did not. One example is the large Swedish cohort study performed by Glatz et al. (36) which compared 33,514 children who had one surgical exposure before age 4 with 159,619 unexposed children, while controlling for factors such as gestational age at delivery, Apgar score at 5 min, and parental educational level. This study measured school grades at age 16 and IQ at age 18. It found no association between anesthesia and later school performance (36). Other studies also found no negative association between surgery/anesthesia and IQ (5), language capabilities (2), or academic performance (37).

Other studies have reported more serious associations between anesthesia/surgery and later-life outcomes such as motor and social linguistic performance (38), expressive language (39, 40), attention deficit hyperactivity disorder (41), diagnosis of developmental/behavioral disorders (40), and parental reporting of behavioral problems (41). Several studies included children with multiple exposures, which seemed to be associated with more severe behavioral problems (42, 43) as well as learning disabilities in reading, written language, and math (44, 45). Additionally, Ing et al. (39) found that children with exposure under 35 min did not differ in scores for total and expressive language scores when compared to unexposed children. However, those with longer than 35 min exposure performed significantly worse.

Given the difficulty of controlling for bias and confounding factors in cohort studies, the preferable form of study is randomized controlled experimentation. Currently, the only randomized controlled trial of anesthesia neurotoxicity has been the GAS experiment (3). Davidson et al. assigned infants who were slated to undergo inguinal herniorrhaphy to either a sevoflurane anesthesia group or an awake regional anesthesia group. The infants were less than 60 weeks’ postmenstrual age with no prior history of general anesthesia and born at more than 26 weeks’ gestation (3). Mean duration of general anesthetic was 54 min. The children were given quantitative intelligence tests at ages 2 (3) and 5 (4), after which the researchers determined that there was no significant difference between the two groups. This study strongly suggests that a short duration of anesthesia early in life has no impact on the IQ score of children (3, 4).

A recent meta-analysis of prospective human studies (35) was conducted including data from the GAS trial as well as the Pediatric Anesthesia NeuroDevelopment Assessment (PANDA) and the Mayo Anesthesia Safety in Kids (MASK) studies (2, 3, 5). These prospective studies were chosen in hopes of minimizing bias and confounding variables. The outcome of this meta-analysis showed no significant difference in general intelligence. However, it did find small but consistent increases in behavioral problems reported by parents.

Overall, anesthesia neurotoxicity is a complicated subject of heated debate. While it has not been possible to prove causation in humans, it would be difficult to conclude that neurotoxic effects of anesthesia, which have been replicated in a wide range of animal models, are of no consequence to humans. Continued research in anesthesia neurotoxicity and neuroprotective strategies is crucial to ensuring the safety of pediatric surgical patients.

## 3. Dexmedetomidine pharmacology

### 3.1. Mechanism of action

Dexmedetomidine may play a role as a neuroprotective agent. It is an agonist of the alpha-2 (ɑ2) adrenoceptor exhibiting ɑ2/ɑ1 selectivity of 1,620:1, which is an almost 8 times greater selectivity compared to clonidine (220:1) (46). Furthermore, there are three isoreceptor subtypes of ɑ2-adrenoceptors: (1) ɑ2A plays a role in sedation, hypnosis, analgesia, sympatholysis, and neuroprotection, (2) ɑ2B induces peripheral vasoconstriction and analgesic effects throughout the spinal cord, and (3) ɑ2C modulates locomotor function, adrenal medullary outflow of adrenaline, as well as cognitive sensory processing and emotional behavioral (e.g., mood) (8).

Dexmedetomidine promotes potassium inflow as an ɑ2 adrenoceptor agonist by opening the K+ rectifying channels, causing cell membrane hyperpolarization to lower the excitability of neuronal cells (8, 46). Dexmedetomidine may also block the flow of calcium into cells, inhibiting phospholipase C activity and its subsequent stimulation of Protein Kinase C (PKC), an enzyme associated with oxidative stress and apoptosis (8).

A lesser-known mechanism of action of dexmedetomidine involves the imidazoline type 2 receptors. Imidazoline receptors are non-adrenergic receptors first found in the ventrolateral medulla (47). In particular, the I2 receptor binding by dexmedetomidine leads to the entry of calcium ions into chromaffin cells. While the precise mechanism of I2 receptors requires further investigation, its interaction with dexmedetomidine is associated with neuroprotection (48).

### 3.2. Pharmacodynamics and physiologic effects

Dexmedetomidine is most commonly used in clinical settings for its sedative effect. The drug creates an unconscious state in a dose-dependent manner that resemble natural sleep, likely achieved through agonism of the central pre- and postsynaptic ɑ2-adrenoceptors to regulate sleep-promoting pathways (49). Dexmedetomidine is unique in its ability to induce “cooperate sedation” during which sedated patients may still be aroused easily without causing respiratory depression (6, 46). Dexmedetomidine is approved by the US Food and Drug Administration for sedation of mechanically ventilated patients in ICU setting up to 24 h; however, studies have demonstrated safe continuous infusion up to 30 days (50, 51). In an intervention review which includes seven studies covering 1,624 participants, Chen et al. found that compared to traditional sedatives (e.g., propofol, midazolam, and lorazepam), long-term sedation using dexmedetomidine in critically ill patients is associated with a 22% lower duration of mechanical ventilation as well as a 14% decrease in length of ICU stay (51). In addition to its use in ICU settings, dexmedetomidine is also a popular choice for procedural sedation such as awake fiberoptic intubation and neurosurgical procedures. The drug has several favorable attributes allowing for successful procedural sedation. For example, it provides adequate analgesia, is associated with a low risk of significant airway obstruction or hypoxia, and reduces hemodynamic instability (52, 53).

Analgesia induced by dexmedetomidine may be related to hyperpolarization of interneurons caused by ɑ2-adrenoceptor activity in central and spinal cords, which in turn prevents peripheral Aδ and C-type nerve fibers from releasing pronociceptive transmitters such as substance P and glutamate (54). Although Angst et al. have found its analgesic efficacy to be lacking with no clinical significance, dexmedetomidine has been associated with opioid-sparing effects demonstrated by lower opioid consumption 24 h after surgery (55, 56).

Lastly, dexmedetomidine is notable for its biphasic hemodynamic response. At high dexmedetomidine levels (e.g., peak plasma levels after bolus injection—usually observed at high concentrations of 1.9–3.2 ng/mL), activation of ɑ2-adrenoceptors on vascular smooth muscles leads to peripheral vasoconstriction and subsequent hypertension, which is followed by reflex bradycardia mediated by carotid or aortic baroreceptors (57). In contrast, a low plasma dexmedetomidine level creates the “hypotensive phase” caused by both vasodilation as well as the sympatholytic effect in which presynaptic ɑ2-adrenoceptors decreases the release of catecholamines through negative feedback (57).

## 4. Dexmedetomidine neuroprotection in animal models

Dexmedetomidine is unique for its many neuroprotective effects, which has been extensively investigated and explored in a variety of animal studies over the past three decades. Here, we will explore five main areas of neuroprotection: (1) Protection against neuroapoptosis, (2) Protection against cerebral ischemia, (3) Protection against neuroinflammation, (4) Preservation of neuroplasticity and synaptic architecture, and (5) Protection against epigenetic modifications on neuronal cells.

### 4.1. Protection against anesthetic-induced neuroapoptosis

Apoptosis, or programmed cell death, is a highly regulated process through which the cell kills itself in a controlled manner in response to internal or external stressors. Anesthetic agents can be a source of apoptosis-inducing stress in neuronal cells. However, several animal studies have shown dexmedetomidine to be capable of countering neuroapoptosis.

In a study by Sanders et al., the neuroprotective effect of dexmedetomidine against isoflurane-induced neuroapoptosis was demonstrated both *in vitro* and *in vivo* using rat models (58). They found that organotypic hippocampal slices from mice pups treated with isoflurane/dexmedetomidine expressed lower levels of caspase-3, an apoptosis-promoting enzyme, compared to the isoflurane/saline control group. Similarly, after exposing 7 day-old rat pups to isoflurane for 6 h and treating with dexmedetomidine or saline, Sanders et al. found reduced levels of apoptosis in the hippocampus, thalamus, and cortex of the experimental group compared to the control; this occurred in a concentration-dependent manner and is likely partially mediated by ɑ2-adrenoceptors (58). Furthermore, rat pups exposed to isoflurane-saline showed a notable deficit in fear conditioning (a measure of long-term memory) at maturity as observed on postnatal day 40, whereas those exposed to air-dexmedetomidine did not; this provided evidence for dexmedetomidine neuroprotection of cognition and behavior on a functional level (58).

Subsequent rodent model studies have reflected similar findings of dexmedetomidine protection through alternate pathways against neuroapoptosis that can be triggered by other types of anesthetics. Perez-Zoghbi et al. showed that dexmedetomidine protected infant rats from sevoflurane-induced neurotoxicity through caspase-3 activation, particularly in the thalamus (59). Using the neonatal rat model, Wang et al.’s findings suggested glutamate regulation as a possible pathway through which dexmedetomidine provides neuroprotection from isoflurane (60). Bao et al. found that dexmedetomidine lowered bupivacaine-induced apoptosis in mouse hippocampal cells, perhaps by promoting the PI3K/AKT pathway and inhibiting the HIF-ɑ/PKM2 axis (61).

### 4.2. Protection in cerebral ischemic injuries

Animal studies have found that dexmedetomidine provides neuroprotection against cerebral ischemia, both when the drug is administered before and after the ischemic event. In a 1991 study, Hoffman et al. injected rats with intraperitoneal saline, high-dose or low-dose dexmedetomidine, then induced incomplete cerebral ischemia by ligating the rats’ right common carotid artery for 30 min (62). The results demonstrated that the hippocampal cells in dexmedetomidine-treated rats had lower levels of histological tissue damage from infarct compared to the control, and that higher dosage offered more protection. In addition, dexmedetomidine-treated rats also had better neurologic outcomes 24–96 h after ischemia, as evaluated by the neurologic deficit score measuring consciousness, walking, limb tone, and pain reflex, among others (62). Finally, plasma catecholamine concentrations during ischemia were significantly lower in the treatment group compared to the control, which suggests that reduced sympathetic activity due to ɑ2-adrenoceptor stimulation by dexmedetomidine may play a role in decreasing ischemic injury (62).

Similar findings were also reflected in a study by Kuhmonen et al. (63). They showed gerbils that received subcutaneous dexmedetomidine injections before and after transient global ischemia (produced by bilateral carotid occlusion) had less damage in the CA1 and CA3 regions of hippocampus as well as hilus of the dentate gyrus compared to the control group. Inhibition of norepinephrine release was also identified as a possible mechanism of dexmedetomidine neuroprotection (63).

While the previously mentioned studies provided evidence of dexmedetomidine-induced neuroprotection when the drug is applied before the ischemic event took place, Maier et al. further demonstrated benefits of postischemic dexmedetomidine administration in rabbits with focal cerebral ischemia (64). The study showed that dexmedetomidine administered to rabbits after focal cerebral ischemia took place offered some protection. Animals treated with the drug had significantly less neuronal damage in the cortex, though not the striatum (64).

### 4.3. Protection from neuroinflammatory processes

Various events ranging from surgical trauma and anesthesia to sepsis may trigger inflammation in the nervous system, initiating the start of many neurological processes including some harmful events. In a series of *in vitro* experiments using BV2 murine microglial cells, Qiu et al. examined the neuroprotective effect of dexmedetomidine in moderating inflammatory factors and mediating microglia cell polarization (65). In their neuroinflammatory cell model, Qiu et al. utilized LPS to stimulate BV2 cells, leading to increased proinflammatory factors (e.g., TNF-ɑ, NO), reduced anti-inflammatory factors (e.g., IL-10), and amplified microglial pro-inflammatory (M1) status; this process is associated with high pERK1/2 expression. However, when BV2 cells were pretreated with dexmedetomidine prior to LPS exposure, the LPS-induced changes were weakened, with a shift towards the microglial anti-inflammatory (M2) state. The overall results suggested that dexmedetomidine promoted microglial M2 polarization, likely by inhibiting pERK1/2, to generate protection for neuronal cells during neuroinflammatory events (65).

The ability of dexmedetomidine to counter neuroinflammation has also been demonstrated *in vivo*. Mei et al. generated a sepsis model by performing cecal ligation and puncture (CLP) on mice, which allowed systemic inflammation to spread to the brain via damaged blood–brain barrier (BBB), thus creating sepsis-associated encephalopathy (66). CLP exposure not only increased proinflammatory cytokines (e.g., TNF-ɑ, IL-6, IL-1β) in the blood and hippocampus, but also led to poor learning and memory in exposed mice 2 weeks after the offending event, as evaluated by fear conditioning and the Barnes maze. In contrast, CLP mice treated with intraperitoneal injection of dexmedetomidine had lower levels of sepsis-induced neuroinflammation, better preserved BBB, and less deficits in learning and memory. Mei et al. identified activation of ɑ2A-adrenoceptors in astrocytes, rather than microglial cells, as the mediating pathway for dexmedetomidine neuroprotection (66).

### 4.4. Preservation of neuroplasticity and synaptic architecture

During the neonatal period in mammals, the developing brain—most notably the dentate gyrus of the hippocampus—undergoes considerable neurogenesis, synaptogenesis, and connectivity which are key processes for learning and memory. In that period, structures in the hippocampus are especially vulnerable to external stressors (e.g., infection, oxidative stress, toxins) that may cause extensive, often irreversible, neuronal damage as well as impairment in neuronal proliferation, migration and plasticity (67, 68). Endesfelder et al. studied the effect of dexmedetomidine on neurogenesis in the dentate gyrus using a hyperoxia-mediated brain injury model in neonatal Wistar rats (67). They found that exposure to hyperoxic conditions significantly lowered the proliferation capacity (as measured by marker PCNA) as well as expression of neuronal markers (Nestin, PSA-NCAM, NeuN) and transcription factors (SOX2, Tbr1/2, Prox1) in hippocampal tissues of neonatal rats. In addition, hyperoxia also reduced regulars (Nrp1, Nrg1, Syp, and Sema3a/f) that are integral in establishing synaptic neurotransmission and structural network (67). However, when the rats were pretreated with a single injection of dexmedetomidine prior to oxygen exposure, the drug upregulated neuronal differentiation, proliferation, migration, and maturation. Dexmedetomidine effectively rescued the developing hippocampus from hyperoxia-induced injuries by improving neuronal plasticity (67).

A study by Lv et al. also demonstrated the protective influence of dexmedetomidine against ethanol-induced toxicity in the hippocampus of neonatal mice (68). Ethanol exposure in neonatal mice inhibited hippocampal neurogenesis and the activity of neural precursor cells, which are responsible for making neurons and providing scaffold to guide the migration of newly generated neurons. Ethanol also activates microglial cells to induce a proinflammatory state, releasing cytokines to create neuroinflammation and neurodegeneration. By pretreating neonatal mice with dexmedetomidine, Lv et al. found a notable attenuation of the ethanol-mediated effects, including the recovery of hippocampal neuronal plasticity and the suppression of inflammation (68).

### 4.5. Protection against epigenetic modifications on neuronal cells

Studies have shown that anesthetic agents may induce neurocognitive changes through epigenetic mechanisms by modifying gene expression of DNA and histone modifying enzymes (69). This in turn promotes neuroinflammation leading to cognitive impairment observed after surgery, i.e., postoperative delirium and/or postoperative cognitive dysfunction (POCD). An example of epigenetic profile-altering mechanisms is DNA methylation (69). A POCD animal model was established by Zhong et al. by treating elderly mice with sevoflurane, which allowed them to study the epigenetic mechanism of anesthesia-induced cognitive impairment. Zhong et al. found that the hippocampus and amygdaloid nucleus of sevoflurane-treated POCD mice had lower levels of global DNA 5′-hydroxymethylcytosine (5hmC) as well as reduced expression of genes associated with neural protection and development (e.g., brain-derived neurotrophic factor (BDNF) and glial cell-derived neurotrophic factor (GDNF)) compared to those of the non-POCD control mice (70). These results fit with the epigenetic model wherein the modification in 5hmC alters the promoters of BDNF and GDNF. In summary, the cognitive deficits seen in POCD mice may be explained by the epigenetic mechanism in which the loss of 5hmC in the brain leads to the reduction of neuroprotective factors.

Expanding upon Zhong et al.’s study, Yang et al. conducted experiments exposing neonatal rats to sevoflurane with or without pre-treatment with dexmedetomidine (71). They found that the rats treated with dexmedetomidine before sevoflurane exposure showed lower levels of seizure-like activity and behavioral deficiencies (evaluated by the Morris Water Maze test and acoustic startle response) compared to those without dexmedetomidine treatment. In addition, the DNA methylation patterns in the hippocampus of dexmedetomidine-treated rats were more similar to the control group (i.e., no sevoflurane exposure), whereas rats exposed to sevoflurane without dexmedetomidine pretreatment had noticeably higher levels of DNA methylation (71). These findings suggest that dexmedetomidine may reduce sevoflurane-induced epigenetic modifications in DNA methylation, thereby attenuating the long-term impairment in neuronal function.

## 5. Postoperative delirium in elderly

Postoperative Delirium (POD) is an adverse peri-operative event that similarly may reflect a possible negative impact of anesthetic agents. POD occurs among 10–60% of the elderly population (72, 73). With the growing number of elderly patients requiring surgery (9, 10, 72, 73), POD is becoming a public health concern.

POD is a syndrome characterized by fluctuating acute cognitive disorder, confusion, restlessness, and agitation after anesthesia (9–11). Clinically, it may present as hyperactive, hypoactive, or mixed during its course (12, 72). POD could be detrimental to patients and their families due to the associated prolonged hospitalization, long-term cognitive decline, higher morbidity and mortality, and high healthcare cost (10, 74, 75). Although POD is known as a serious complication, currently there is no definite preventive measure (76).

The mechanism of delirium is multifactorial and not well understood (9, 11, 12, 77). POD could be associated with poor pain management, hypoxia, anesthetic technique, opioid use, inflammation, and neurotransmitter imbalances, especially dopamine and acetylcholine (9, 12, 74). Among these, the two leading theories of delirium are the disorders of inflammation and inadequate cerebral perfusion. Systemic inflammation causes secretion of cytokines which acts on the blood brain barrier while suppressing neuronal excitability and connectivity. Inadequate cerebral perfusion can result from low cardiac output, hypotension, vascular autoregulation, and secondary cellular metabolic stress (77).

Dexmedetomidine has properties that may blunt the impact of several presumed mechanisms of POD. The drug has been employed in the context of elderly patients requiring mechanical ventilation to reduce the incidence of delirium (73, 78). A possible role of dexmedetomidine in POD has yet to be fully defined. However, its use may contribute in several ways.

Administering dexmedetomidine may reduce delirium by lowering post-operative pain and opioid use (9, 12). It provides protection against hypoxia due to hypoventilation during the recovery period which may reduce delirium (9). Clinical evidence also indicates that administering intraoperative dexmedetomidine prevents over-secretion of cytokines during and after surgery (12).

Some randomized studies show that intraoperative use of dexmedetomidine may reduce POD in elderly patients (9, 79–82) while other studies contradict this finding (72, 83–85). The surgical settings studied include cardiac (81, 82, 84, 85), orthopedic (9, 76, 80), non-cardiac major surgeries (11, 12, 72), and cancer surgeries (79). Sample sizes vary from 115–798. Different scales to assess POD such as RAAS, MMSE, CAM, CAM-ICU were used. Additionally, the studies used different dosages, administration modes, and time (Table 1). Dosages varied from 0.1 mcg/kg/h to 0.6 mcg/kg, depending on administration mode and time. Some randomized trials used a loading dose followed by a continuous infusion (11, 12, 81, 82). In the Sedation Practice in Intensive Care Evaluation (SPICE III) trial, researchers studied the infusion rates and use of dexmedetomidine and propofol for sedation in the intensive care unit. Researchers found that patients 65 years of age sedated with dexmedetomidine supplemented with titrated propofol had decreased adjusted 90 day mortality, and increased mortality was found to be associated with increasing dexmedetomidine infusion rates (86).

**Table 1:** Basic features of randomized clinical trials.

First author	Study population	Number	Dex dosage and administration time	Scales used	Anesthesia induction	Anesthesia maintain	Conclusion
Dong Jun Kim	Orthopedic	115	100 mg/mL was administered at 0.4 μg kg − 1 h − 1(DEX)or Normal saline (0.1 mL kg − 1 h − 1)-Intra-operative	VAS, RSAS	Midazolam 30 min before anesthesia. Propofol, Sevoflorane, remifentanil, and rocuronium	Sevoflurane or propofol and remifentanil	Dexmedetomidine lowered the emergence agitation
Hong Hong	Orthopedic	712	Postoperative patient-controlled intravenous administration (IV) of either dexmedetomidine 200 μg or 0.9% saline and 200 μg sufentanil, diluted with 0.9% saline to 160 mL. The pump was programmed to deliver 2-ml boluses with a lockout interval of 8 min and a background infusion of 1 mL.h − 1.	RASS, Cam, Cam-ICU	Midazolam (1–3 mg), propofol or etomidate and sufentanil or remifentanil.	Propofol infusion, sevoflurane and/or nitrous oxide inhalation, and sufentanil or remifentanil	Low dose dexmedetomidine and supplemental intravenous analgesia did not reduce delirium. Although, it improved analgesia and sleep quality without aggravating adverse events.
Ting Huyan	Lung cancer	346	Dexmedetomidine (0.5 μg/kg) was administered in 20 min before the beginning of the operation followed by a continuous IV infusion at a rate of 0.1 μg/kg/h during the intraoperative period. The drug infusion was stopped 30 min before the end of the operation.	Intensive Care Delirium Screening Checklist (ICDSC)	Etomidate, Fentanyl, Rocuronium	Propofol, remifentanil and cisatracurium, sufentanil and atracurium cis-benzenesulfonate	Pre-operative and intra-operative administration of dexmedetomidine can lowered the incidence of delirium. Dexmedetomidine also lowered pain and improved sleep quality.
Xiaoyuan Sui	>4 h anesthesia laparotomy- non-cardiac	N/A	Dexmedetomidine hydrochloride is injected with a micro infusion pump within 15minutes before anesthesia induction with pre-injection loading dose (0.5μg/kg), followed by continuous infusion at a rate of 0.3μg/kg/h	MMSE VAS	Midazolam, etomidate, sufentanil, cisatracurium	Propofol and remifentanil (study was not finished)	This study will find out the efficacy and safety of dexmedetomidine in lowering POD
Stacie Deiner	Non-cardiac major surgeries	404	Dexmedetomidine infusion (0.5 μg/kg/h) during surgery and up to 2 h in the recovery room.	CAM, CAM ICU, MMSE	Avoided benzodiazepines and nitrous oxide, any other induction agents were permissible	Propofol, sevoflurane or both	Intra-operative infusion of dexmedetomidine does not decrease POD
Yuqin Lv	Hip re-placement	327	The treatment group was treated with 0.1 μg/kg/h DEX, while the placebo group was administered with an equal amount of normal saline solution intravenously within 72 h following THA surgery.	CAM	No pre-medications, along with a standard preoperative evaluation and the same general anesthesia protocol using IV midazolam (1–2 mg) and fentanyl (50–100 mg) for preoperative sedation	3–6 mg/kg IV fentanyl	Dexmedetomidine can reduce POD
Bo-Jie Wang	Non-cardiac- >2 h	620	A loading dose dexmedetomidine (0.6 μg/kg) will be administered 10 min before anaesthesia induction, followed by a continuous infusion at a rate of 0.5 μg/kg/h until 1 h before the end of surgery.	CAM, CAM-ICU	Intravenous sufentanil and propofol	Intravenous sufentanil, propofol and inhalation of a 1:1 nitrous oxide–oxygen mixture. Rocuronium and/or cisatracurium are administered for muscle relaxation.	The study is investigating if dexmedetomidine use during general anesthesia can lower POD
Balachundhar Subramaniam *et al*	CABG/valve surgery in US	120	4 groups of patients: IV acetaminophen plus dexmedetomidine, IV acetaminophen plus propofol, Placebo plus dexmedetomidine and Placebo plus propofol. Dexmedetomidine group received an IV bolus dose of 0.5 to 1 μg/kg during chest closure, followed by a maintenance infusion of 0.1 to 1.4 μg/kg per hour. The IV propofol group received a maintenance dose of 20 to 100 μg/kg per minute. With placebo vs. acetaminophen administered within 1 h of ICU admission and thereafter every 6 h for 8 doses	CAM/CAM-ICU	Not found		Post-operative intravenous (IV) acetaminophen combined with IV propofol or dexmedetomidine reduced POD
Alparslan Turan	Cardiac	798	Either dexmedetomidine infusion or saline placebo started before the surgical incision at a rate of 0·1 μg/kg/ h then increased to 0·2 μg/kg/ h at the end of bypass, and postoperatively increased to 0·4 μg/kg/h, which was maintained until 24 h.	CAM-ICU	Midazolam, thiopental, etomidate, propofol, and sufentanil or fentanyl, or both, and	With volatile anaesthetics and a non-depolarising muscle relaxant	Dexmedetomidine infusion before surgical incision and continued for 24 h post-operatively did not decrease atrial fibrillation and POD
Mona Momeni	cardiac	420	Propofol infusion and dexmedetomidine (0.4 μg kg-1 h-1) or a propofol infusion and saline 0.9% (placebo group).	MMSE, CAM, CAM-ICU	Midazolam 0.03–0.06 mgkg^−1^, sufentanil 0.30.5μgkg^−1^, ketamine 0.3–0.5 mg kg^−1^, and a bolus dose of propofol. A continuous infusion of sufentanil at a rate of 0.5–0.8 μg kg^−1^ h^−1^ was administered for intraoperative analgesia.	Sevoflurane. Sevoflurane was continued during the CPB period.	The study does not recommend supplementing post-operative propofol with dexmedetomidine to reduce POD
George Djaiani	cardiac	185	Dexmedetomidine 0.4 μg/kg bolus followed by 0.2 to 0.7 μg kg^−1^ h^−1^ infusion or propofol 25 to 50 μg kg^−1^ h^−1^ infusion	CAM, CAM-ICU	10 to 12 μg/kg fentanyl, 0.5 to 2 mg/kg propofol, and 0.15 mg/kg pancuronium and	Maintained with 0.5 to 2.0% isoflurane.	Dexmedetomidine reduced incidence, delayed onset and shortened duration of POD

Despite these limitations, there is evidence supporting the role of dexmedetomidine. A meta-analysis concluded that dexmedetomidine can decrease the incidence of POD in adult cardiac and non-cardiac patients (87). A systemic and meta-analysis by Shen et al. (73), found that prophylactic use of dexmedetomidine may significantly reduce the risk of POD in elderly patients presenting for non-cardiac surgery. Sequential analysis showed that the information size was sufficient to support a role for dexmedetomidine in the prevention of POD.

Another systematic review and meta-analysis by Qin et al. examined 13 trials involving 4,015 patients. The analysis suggests that perioperative dexmedetomidine could decrease POD in the elderly presenting for non-cardiac surgeries, as well as use of the drug was associated with increased risk of bradycardia and hypotension (88). A meta-analysis by Pasin et al. examined 14 randomized studies and 3,029 patients and concluded that dexmedetomidine could help to reduce POD in critically ill patients in ICU (78). A different meta-analysis by Liu et al. compared dexmedetomidine versus propofol sedation after cardiac surgery and showed that dexmedetomidine might reduce POD and shorten the duration of intubation (89). After the high-risk biased trials were excluded, a systematic review by Patel et al. included 30 trials and 4,090 adult patients who underwent cardiac surgery. Researchers concluded that administration of perioperative dexmedetomidine was not associated with lowering the incidence of POD. However, their pooled analysis showed the potential benefit of dexmedetomidine decreasing the duration of POD as well as the hospital and ICU length of stay. It was also found that dexmedetomidine use lowered the occurrence of respiratory complications and acute kidney injury (77).

In general, there is a great deal of heterogeneity in clinical studies related to the use of dexmedetomidine to prevent POD. Existing studies may assist for specific clinical choices but there is insufficient evidence to support broad conclusions and application. Overall, there are numerous areas for thoughtful and impactful research.

## 6. Emergence delirium and emergence agitation in pediatric patients

As opposed to the elderly, there is broad clinical use of dexmedetomidine including for treatment and prevention of emergence delirium in the pediatric population. Indications include treating agitation, withdrawal symptoms, and delirium (90).

Of note, numerous studies support an impact on delirium. Shi et al. performed a double-blind randomized trial and found the use of intravenous (IV) dexmedetomidine 0.5 μg/kg in pediatric patients after tonsillectomy was beneficial in preventing ED when patients were anesthetized with sevoflurane based on the pediatric anesthesia emergence delirium scale (PAEDS) (91). Tsiotou et al. completed a double-blind, randomized study examining pediatric patients undergoing tonsillectomy with or without adenoidectomy using total intravenous anesthesia (TIVA) with propofol. They studied the presence of ED with and without dexmedetomidine. The presence of ED was measured using the Watcha scale, and the study found a significant decrease in the incidence and severity of ED in the group treated with dexmedetomidine compared to TIVA alone without prolonging extubation time (92).

While IV dexmedetomidine treatment has proven to be favorable, alternate routes of delivery also offer advantages in treating the pediatric population, such as via intranasal (IN) administration. Wang et al. performed a randomized clinical trial comparing the use of IN dexmedetomidine and oral midazolam for premedication in pediatric dental patients undergoing general anesthesia. Based on the PAEDS, they showed a significantly lower number of children experiencing ED in the dexmedetomidine group versus the midazolam group (93). The benefit of IN dexmedetomidine was also reflected in a study by Shen et al. In a randomized clinical trial, the researchers compared the incidence of ED in pediatric patients undergoing tonsillectomy and adenoidectomy when treated with IN dexmedetomidine, IN midazolam, or control IN normal saline. They found a significant decrease in incidence of ED in the IN dexmedetomidine group compared to the IN midazolam and control groups (94).

Dexmedetomidine has been applied in the context of regional anesthesia including retrobulbar administration. In a randomized, double-blind study, Varsha et al. compared caudal epidural and ultrasound-guided ilioinguinal-iliohypogastric block with bupivacaine and dexmedetomidine for local anesthesia after general anesthesia in pediatric patients undergoing inguinal hernia repair (95). Researchers assessed ED using PAEDS and found no difference in incidence of ED between the two groups (95). On the other hand, Ye et al. performed a randomized control study to investigate the effect of retrobulbar dexmedetomidine in pediatric vitreoretinal surgery (96). Patients were grouped in one of three ways: retrobulbar block with ropivacaine plus dexmedetomidine, retrobulbar block with ropivacaine alone, and no retrobulbar block with general anesthesia alone. The study found that emergence agitation was significantly lower in the retrobulbar dexmedetomidine-treated group when compared to the ropivacaine only group (96).

Dexmedetomidine also has a role in treating emergence agitation (EA) in the pediatric population. Shafa et al. compared the effectiveness of two distinct doses of dexmedetomidine (1 μg/kg and 2 μg/kg) versus placebo in treating agitation, which was measured using PAEDS in pediatric patients undergoing adenotonsillectomy (97). The researchers found that the use of dexmedetomidine at both doses was associated with a significant reduction in agitation upon emergence (97). There are also further studies comparing dexmedetomidine bolus versus continuous infusion and the effect on EA.

Begum et al. compared a bolus of dexmedetomidine (0.4 mcg/kg over 10 min) with low-dose dexmedetomidine infusion (0.4 mcg/kg/h) in pediatric patients undergoing sevoflurane general anesthesia. They found that PAEDS scores were less suggestive of delirium and the incidence of EA was significantly lower in the bolus group compared with the low-dose infusion group. Furthermore, the bolus group had a reduced postoperative opioid consumption and bolus dosing did not prolong patients’ length of stay in the post anesthesia care unit (98).

In light of the presumed mechanisms of action and the large number of trials it can be inferred that dexmedetomidine may play a role in blunting physiology that produces delirium. This would also suggest a possible impact on the theoretical risk of neurotoxicity.

## 7. Conclusion

There are numerous theoretical and practical considerations related to possible negative impact of anesthetic agents on the central nervous system. These potentially include neurotoxicity and delirium. Dexmedetomidine is a widely employed agent with properties and a growing body of evidence to suggest a role in neuroprotection in pediatric and adult populations. Greater understanding of this possible role of dexmedetomidine is a fertile area for future research.

Existing studies related to neuroprotection are limited by heterogeneity that limits broad applicability. Many of the studies reviewed included pediatric patients undergoing elective procedures at low risk, ranked as American Society of Anesthesiologists (ASA) Physical Status Classification System class I or II (91, 92, 94, 95, 97, 98). Future studies including high risk patients undergoing major surgeries and emergency surgeries with higher ASA classes may be included to contribute to the data and research in the application of dexmedetomidine for neuroprotection in the general population. In light of the scope of practice and clinical concerns related to neurotoxicity, there is clear translational value to this type of information. While current evidence may not be strong enough to conclude that dexmedetomidine is purely neuroprotective, its clinical value in high risk patients is worth exploring.

## Author contributions

AT and AW literature review and preparation of the first draft of the manuscript, critical review, and editing of the manuscript. JM and AK literature review and preparation of the first draft of the manuscript. ZJ design and conceptualization of the work, critical review, and editing of the manuscript. RM, VT, and SB critical review, revisions, mentorship, and editing of the manuscript. All authors contributed to the article and approved the submitted version.

## Conflict of interest

The authors declare that the research was conducted in the absence of any commercial or financial relationships that could be construed as a potential conflict of interest.

## Publisher’s note

All claims expressed in this article are solely those of the authors and do not necessarily represent those of their affiliated organizations, or those of the publisher, the editors and the reviewers. Any product that may be evaluated in this article, or claim that may be made by its manufacturer, is not guaranteed or endorsed by the publisher.
